# Room-Temperature Reduction of Graphene Oxide in Water by Metal Chloride Hydrates: A Cleaner Approach for the Preparation of Graphene@Metal Hybrids

**DOI:** 10.3390/nano10071255

**Published:** 2020-06-28

**Authors:** Patrick. P. Brisebois, Ricardo Izquierdo, Mohamed Siaj

**Affiliations:** 1 Department of Chemistry, Université du Québec à Montréal, NanoQAM/QCAM, Montreal, QC H3C 3P8, Canada; patb.office@gmail.com; 2 École de Technologie Supérieure, Université du Québec, Montreal, QC H3C 1K3, Canada; ricardo.izquierdo@etsmtl.ca

**Keywords:** graphene oxide, chloride hydrates, ^13^C NMR spectroscopy, metal oxide

## Abstract

Headed for developing minimalistic strategies to produce graphene@metal hybrids for electronics on a larger scale, we discovered that graphene oxide (GO)-metal oxide (MO) hybrids are formed spontaneously in water at room temperature in the presence of nothing else than graphene oxide itself and metal ions. Our observations show metal oxide nanoparticles decorating the surface of graphene oxide with particle diameter in the range of 10–40 nm after only 1 h of mixing. Their load ranged from 0.2% to 6.3% depending on the nature of the selected metal. To show the generality of the reactivity of GO with different ions in standard conditions, we prepared common hybrids with GO and tin, iron, zinc, aluminum and magnesium. By means of carbon-13 solid-state nuclear magnetic resonance using magic angle spinning, we have found that graphene oxide is also moderately reduced at the same time. Our method is powerful and unique because it avoids the use of chemicals and heat to promote the coprecipitation and the reduction of GO. This advantage allows synthesizing GO@MO hybrids with higher structural integrity and purity with a tunable level of oxidization, in a faster and greener way.

## 1. Introduction

Graphene oxide (GO) is regarded as a major precursor of graphene-based metal (M) and metal oxide (MO) particle nanocomposites, important building blocks for electronic and electrochemical devices [1,2,3,4,5]. The presence of oxygen atoms and metal particles in the composite can change dramatically the physical, electronic and chemical properties of graphene-based devices [4]. GO@M/MO materials have shown great promises in various hi-tech application fields such as paramagnetic agents for magnetic resonance imaging (M = Fe) [6,7,8], capacitive electrodes for lithium batteries (M = Fe, Co, Sn) [9,10], supercapacitors (M = Cu, Ti, Mn) [11,12,13,14], photocatalysts (M = Ti, Sn, W) [15,16,17,18,19,20], electrocatalysts (M = Au, Pd, Pt) [21,22,23], catalysts for chemical transformation (M = Au, Pt) [24,25], electrodes (M = Ti, Zn) [26,27], conductive transparent films (M = Cu) [28], sensing [29,30] (M = Sn, Pd, Zn) [31,32,33,34,35], water remediation (M = Fe) [36,37], molecular separation [38] and antibacterial nanocomposites (M = Ag, Cu, Zn, Mn, Se) [39,40]. According to previous reports [41], C–OH on GO can easily react with metal cations to form C–O–M.

GO has the ability to make chemical and physical interactions with different metal or metal oxide through others oxygenated defects as well [4]. A recent study showed that most of C–O–M bonds are from the reaction between epoxy and metal oxide and/or hydroxide, while only a part of C–O–M bonds could be explained by GO’s carboxyl (C=O) and hydroxy (C–OH) groups. Evidence was mainly collected from infrared (FTIR) and X-ray photoemission spectroscopy (XPS) data.

Several methods [1,2] are available to prepare GO@M/MO composites on a gram scale using the solution mixing method [42], the sol–gel method [43], the hydrothermal/solvothermal method under pressure and heat [38], by self-assembly [44] or conjugation [30] with preformed particles, by spontaneous redox reaction between metal and GO in solution [31] or by dry mechanomecanical metal reduction of GO [45]. When mixed together in solution, metal ions of different nature can be coprecipitated to form binary metals systems on GO [46,47,48]. The usual solvents used in the precipitation method are water, water–ethanol [42] mixtures or dimethylformamide (DMF) [32]. To achieve the in-situ covalent binding or loading of metal and metal oxide nanoparticles (NPs) on GO’s surface, heat, microwave irradiation [23] or UV irradiation [49] are always used in combination with chemicals [1,2,4,50]. The most employed chemicals used in these transformations are: hydrazine [46], sodium borohydride [51] or glucose [52] as reducing agents; sodium or potassium hydroxide and hydrochloric acid as pH adjusters [46] and surfactants [43]. Noteworthy, all these methods lead to GO with a higher degree of reduction, hence called reduced graphene oxide (rGO).

Unfortunately, approaches using chemicals and extreme conditions entail structural damages and result in the breaking of graphene oxide sheets into smaller fragments [52]. Every time GO is mixed with a reagent containing heteroatoms, some will likely be trapped and will remain in the composite as an impurity (doping). Furthermore, it is very difficult to control the size and shape of the NPs during their formation at high temperature. Those factors have a great impact on the efficacy of the material for a specific application because they affect the mechanical properties and the electronic bandgap of the material [6,53]. The key challenge to improve the performance of the GO@M/MO composite is to develop a milder synthesis to avoid these problems [54,55,56].

Herein, we addressed these issues and have found a way to avoid the use of any chemicals, heat or irradiation to synthetize high purity GO@MO hybrids with minimal defects. We developed a minimalistic very mild method using only GO and concentrated aqueous solutions (1 M) of metal chloride hydrates (SnCl_2_·2H_2_O, FeCl_2_·4H_2_O, ZnCl_2_·2H_2_O_,_ AlCl_3_·6H_2_O or MgCl_2_·6H_2_O), which have a natural acidic pH. Our observations show the room temperature formation of metal oxide nanoparticles decorating the surface of GO, while our carbon-13 solid-state nuclear magnetic resonance (13C SS-NMR) data show a moderate reduction of GO compared to the starting material. All our materials were further characterized using transmission electronic microscopy (TEM), thermogravimetric analysis (TG/DTGA), Fourier transform infrared (FTIR), X-ray photoelectron (XPS), energy-dispersive X-ray (EDS) and Raman spectroscopy techniques, giving a hint on how metal oxides can form in the absence of external stimuli.

## 2. Experimental

### 2.1. Synthesis of Graphene Oxide

Graphene oxide was prepared using a modified procedure of the Hummer’s method developed by Tours and collaborators [57]. Briefly, Graphite (3 g) was mixed with H_2_SO_4_ (360 mL) and H_3_PO_4_ 85% (40 mL). Then, the mixture was heated to 50 °C and stirred while KMnO_4_ (18 g) was added slowly over 5 min. Short periods of ultra-sonication (4×) were applied every hour (15 min) over the course of the reaction (4 h) to favor exfoliation. Once the reaction was completed, the thick mixture was allowed to cool down to room temperature and was neutralized slowly over a mixture of ice (600 g) and hydrogen peroxide 30% (10 mL) with strong evolution of gas. The crude GO was recovered using centrifugation (10,000 RPM, 2 h) and the brown solids were washed successively with 200 mL of water, 200 mL of HCl 10% and 500 mL of anhydrous ethanol (2×). The material was finally precipitated in anhydrous ether (1000 mL) and recovered using vacuum filtration over a Teflon^®^ membrane (0.45 μm). The light brown material was dried under vacuum (24 h) below 40 °C yielding 5.9 g of a mixture of graphene/graphite oxide.

The crude material (5.9 g) containing a mixture of graphite and graphene oxide was suspended in NanoPure water (1 L) using stirring (12 h) and sonication (60 min) until complete homogenization. Some unreacted graphitic particles and thick multilayers were still visible in the suspension and were removed by centrifugation (5000 rotation per minute (RPM), 30 min). The large intact graphene oxide flakes were recovered using centrifugation at 10,000 RPM (2 h) and the smaller flakes were eliminated in the remaining liquid. The portion containing the intact sheets (10,000 RPM) was recovered as a hydrogel and was freeze-dried under vacuum to remove water completely. The solid material was dried below 40 °C under high vacuum (24 h) and stored in a sealed desiccator over P_2_O_5_ for one week, yielding 5.2 g of dehydrated graphene oxide.

### 2.2. Preparation of Aqueous Suspension of GO

Dehydrated graphene oxide (1 g) was suspended in NanoPure water (200 mL) using sonication (30 min × 2) and stirring (12 h × 2) at room temperature resulting in an aqueous homogenous suspension of 5 mg mL^−1^.

### 2.3. Preparation of 2 M Aqueous Solution of Metal Chloride

Metal chlorides (0.4 mol) such as SnCl_2_·2H_2_O, FeCl_2_·4H_2_O, ZnCl_2_, AlCl_3_·6H_2_O and MgCl_2_·6H_2_O were dissolved separately in NanoPure water (200 mL) using stirring (1 h) at room temperature resulting in a 2 M aqueous solution. In the case of SnCl_2_·2H_2_O and FeCl_2_·4H_2_O, a saturated solution was obtained and used as prepared without filtration of the insoluble particles.

### 2.4. Synthesis of GO@MO Hybrids

GO suspension (200 mL, 5 mg mL^−1^) was added rapidly in a beaker over the 2 M aqueous solution of metal chlorides (200 mL) (e.g., SnCl_2_·2H_2_O, FeCl_2_·4H_2_O, ZnCl_2_·4H_2_O, AlCl_3_·6H_2_O or MgCl_2_·6H_2_O), resulting in a final suspension (400 mL) with a concentration of 1 M in metal ions and 2.5 mg mL^−1^ in GO. The mixture was stirred vigorously for 1 h at room temperature (24 °C) and atmospheric pressure (standard conditions) using magnetic stirring. During the reduction, the color of GO changed from orange to light brown and the pH of the solutions was evaluated using paper strips. After the reaction, the hybrid materials were recovered using centrifugation (10,000 RPM, 2 h). The supernatant liquid containing metal ions was collected aside and recycled for a further reaction with GO. Then, the materials were washed successively with 200 mL of HCl 5% (3×) and with 200 mL of deionized water (3×) to remove free particles of metal oxide. The brown materials were freeze-dried under vacuum to remove water completely. Then, they were dried below 40 °C under high vacuum (24 h) and stored in a sealed desiccator over P_2_O_5_ for one week prior to analysis, yielding 0.8–1.2 g of dehydrated GO@MO composites.

### 2.5. Characterization Methods

^13^C-SS MAS-NMR spectra were recorded with Bruker Avance III HD spectrometer (Milton, ON, Canada) operating at frequencies of 150.874 MHz for ^13^C and 599.84 MHz for ^1^H using a 4-mm magic-angle spinning (MAS) double resonance probe and a zirconium oxide rotor for NMR analysis. Magic angle spinning was performed at a spinning frequency of 12.5 kHz. Typically, 100 mg of GO material or hybrid was used in the sample and 4096 scans were recorded (or otherwise specified) to achieve desired spectral resolution. Direct Pulse-^13^C-SS MAS-NMR spectra were recorded using a broadband proton decoupling at a RF field of 87.5 kHz during acquisition, with a spectral width of 75 kHz, a 90° pulse length of 3.3 µs, a 20 s recycle delay and an acquisition time of 20 ms. All spectra were collected in duplicate at room temperature (23 °C). Data were analyzed using the Mestrenova^®^ software V6.0 (Mestrelab Research, Santiago de Compostela, Spain). Exponential line broadening functions of 50 Hz were applied to the MAS spectra and chemical shifts were referenced relatively to adamantane (38.25 ppm).

FTIR/ATR data were acquired on a Nicolet smart iTR 6700 spectrometer (Thermo-Nicolet, Madison, WI, USA). Dry solid samples of GO and derivatives were used directly for ATR analysis without sample preparation. Data were analyzed using OMNIC^®^ software (Thermo-Nicolet, Madison, WI, USA). The chemical composition of the surface was investigated by X-ray Photoelectron Spectroscopy, using a PHI 5600-ci spectrometer (Physical Electronics, Eden Prairie, MN, USA). The main XPS chamber was maintained at a base pressure of <8 × 10^−9^ Torr. A standard magnesium X-ray source was used to record survey spectra (1253.6 eV, 10 min) and high-resolution spectra, without charge neutralization. The detection angle was set at 45° with respect to the normal of the surface and the analyzed area was 0.005 cm^2^ (aperture 4). A peak fitting process was performed where the linear background was first deducted from the original XPS data, after which the spectra were aligned using the C–C bond energy (284.5 eV) by using the C 1s of a grounded Highly Oriented Pyrolytic Graphite (HOPG) layer and then fitted using a Gaussian–Lorentzian lineshape. Raman spectroscopy (Renishaw, inVia Reflex, West Dundee, Il, USA) was performed with 514.5 nm laser excitation at a power of 10 mW. TGA/DTGA analysis were performed using typically 5 mg of dry material with a thermogravimetric analyzer (TGA Q500/Discovery MS, New Castle, DE, USA) under helium (He) with a heat ramp of 5 °C/min. The pH of the solutions was measured using color-fixed indicator strips (Macherey-Nagel inc, Bethlehem, PA, USA).

## 3. Results and Discussion

**Figure 1:**
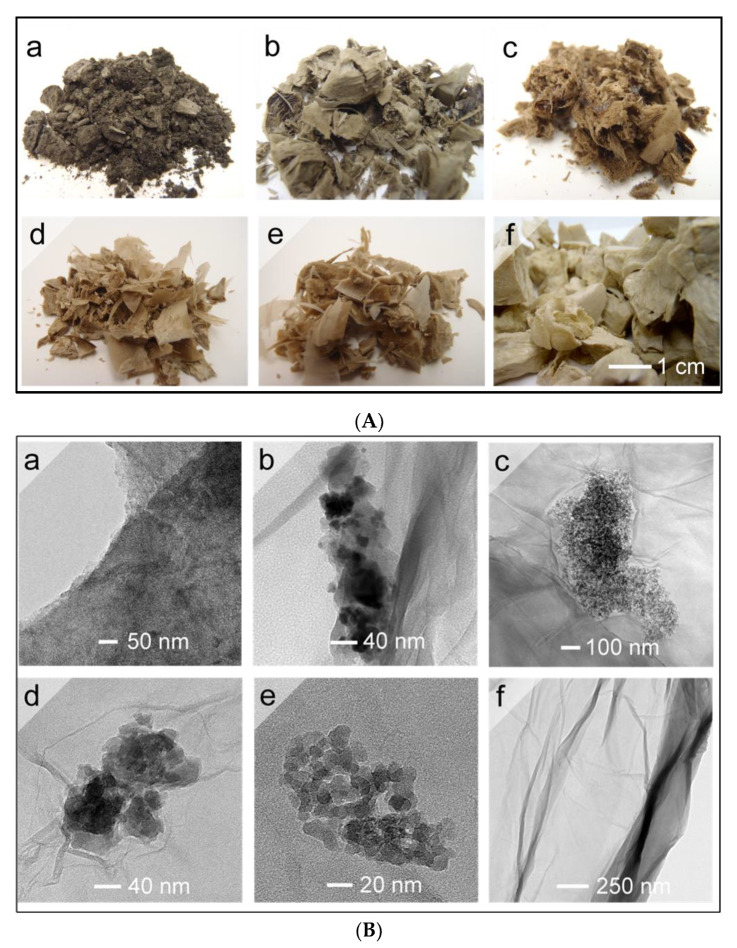
(**A**) Optical images; and (**B**) TEM images of: (**a**) GO-Sn; (**b**) GO-Fe; (**c**) GO-Zn; (**d**) GO-Al; (**e**) GO-Mg; and (**f**) GO materials.

The synthesis of graphene oxide was achieved via the oxidation of graphite using a modified Hummer’s method [57,58]. After the oxidation step, golden flakes of GO were suspended in deionized water and reacted with metal salts solutions (SnCl_2_·2H_2_O, FeCl_2_·4H_2_O, ZnCl_2_·2H_2_O_,_ AlCl_3_·6H_2_O and MgCl_2_·6H_2_O) under ambient conditions. In the mixture, the metal ions interact with water and GO to form the hydroxide and some hydrochloric acid is produced in situ. Therefore, the aqueous mixtures were acidic, and their pH values were evaluated as 1.2, 1.5, 5.4, 2.0 and 5.3, respectively, for GO-Sn, GO-Fe, GO-Zn, GO-Al and GO-Mg suspensions. After 1 h in the acidic solution, the GO suspensions showed no flocculation or aggregation. This indicates that the reaction products still had a fair number of polar groups attached to their structure, which kept the material in suspension through H bonding with the oxygenated groups of GO. The solid hybrid materials were collected by centrifugation at high speed (10,000 RPM). The excess of reagents was washed away with a 5% HCl solution followed by a copious amount of water to eliminate impurities and free particles not adsorbed on GO. The solution containing unreacted dissolved salts was recuperated in the centrifugation step and recycled for a further batch by simply adding the right amount of metal chloride hydrates to reach the starting concentration (2 M). After lyophilization and proper dehydration, flakes of GO-Sn, GO-Fe, GO-Zn, GO-Al and GO-Mg ranging from light orange to light brown were recuperated (Figure 1A).

Since reduction can make a great change in the structure of GO, microscopic observation was used to judge the reducing effect of different metal ions on GO [52,53]. TEM images of GO and of the as-dried GO-MO composites are used to show the structure and property changes of GO after reduction. First, Figure 1B shows the formation of spherical NPs on the surface of GO. Noteworthy, the general aspect of GO sheets in GO-Sn, GO-Fe, GO-Zn, GO-Al and GO-Mg remained intact after the reaction. Upon contact with GO, the dissolved metal salts (e.g., SnCl_2_·2H_2_O, FeCl_2_·4H_2_O, ZnCl_2_·2H_2_O_,_ AlCl_3_·6H_2_O and MgCl_2_·6H_2_O) reacted with the oxygenated groups of graphene oxide to produce a complex with the general formula [GO-M^n+^] [10,52]. This intermediate may facilitate the reduction of GO either by electron transfer [12] (Sn^2+^ and Fe^2+^) or acidic solvolysis (Zn^2+^, Al^3+^ and Mg^2+^) [52]. Meanwhile, spontaneous formation of MO NPs was observed on the surface of GO with the approximate particle diameter of 10–40 nm. Noteworthy, the amount of MO NPs found in the hybrid materials was higher when stannous chloride and iron chloride were used as reducing agents. In the case of GO-Sn hybrid, the entire surface of GO was covered uniformly with small NPs of around 10 nm and the surface had a darker appearance. Upon magnification, a moderate number of larger NPs was observed for GO-Fe. Both elements (Sn and Fe) were easily identified using EDS (Figure S1 spectroscopy. GO-Sn composite showed a series of EDS peaks at 3.05, 3.27, 3.44, 3.67, 3.91, 4.13 and 4.38 KeV. GO-Fe composite had characteristic peaks at 6.40 and 7.06 KeV, respectively, for Fe 2p and Fe 3p [59]. In contrast, GO-Zn, GO-Al and GO-Mg composites showed by TEM only few NPs aggregated under islands randomly dispersed on the surface of GO. Therefore, Zn, Al and Mg elements could not be detected with accuracy using EDS. The amounts of metal contained in the GO-MO composites were evaluated by XPS (atomic composition) as 6.8%, 1.0%, 0.2%, 0.2% and 0.3%, respectively, for elements Sn, Fe, Zn, Al and Mg (Table 1).

**Figure 2:**
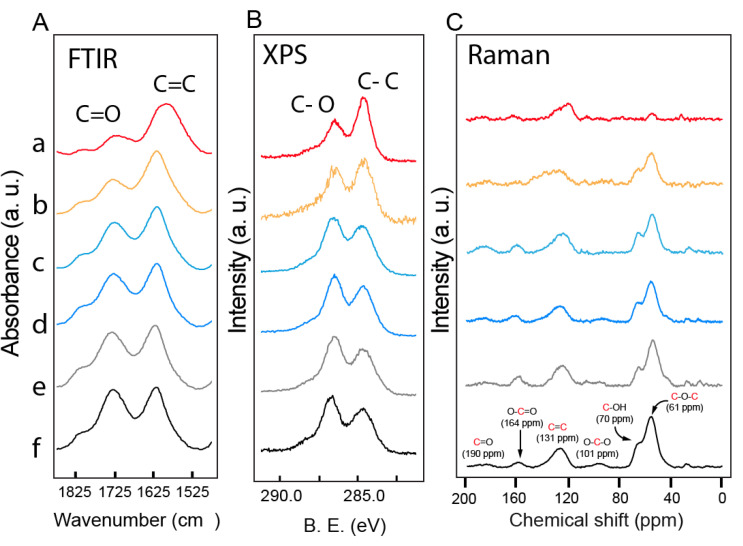
(**A**) FTIR; (**B**) XPS C1s; and (**C**) Raman spectrum of: (**a**) GO-Sn; (**b**) GO-Fe; (**c**) GO-Zn; (**d**) GO-Al; (**e**) GO-Mg; and (**f**) GO materials. ^13^C direct pulse solid-state NMR (MAS) spectrum of GO-Sn, GO-Fe, GO-Zn, GO-Al, GO-Mg and GO materials (speed of rotation: 12.5 kHz).

Table 1 shows the atomic composition of the GO-MO composites (excluding hydrogen atom). The graphene oxide used for the preparation of the hybrids has a C/O ratio of 1.8, which indicates a high degree of oxidation of the starting material [57]. The obtained GO-Fe, GO-Zn, GO-Al and GO-Mg complexes have a slightly higher C/O ratio of around 1.9, which confirms the very mild aspect of the reduction of GO. Stannous chloride in solution in the presence of hydrochloric acid is a powerful reducing agent and can reduce GO [60]. For this, GO-Sn composite should be reduced considerably compared to GO but XPS data (Table 1) show surprisingly lower C/O value (1.6) compared to expectations. Overoxidation of GO was ruled out. This can rather be explained by a significative amount of Sn oxide in the composite increasing the intensity of the O 1s peak (534 eV) considerably. Because XPS C/O ratio considers the total amount of oxygen atoms in the composite (GO + NPs), it is useless to estimate the level of reduction for GO-MO structures. Infrared spectroscopy (Figure 2A) is well-suited for the monitoring of the reduction process because GO exhibits a well-defined elongation band at 1720 cm^−1^ corresponding to the carbonyl region (C=O) and a band at 1588 cm^−1^ corresponding to C=C elongation (graphitic material) [6,43]. The relative ratio of those two signals is representative of the oxygen-containing functional groups vs. the amount of graphitic material found in GO. Upon reduction in contact with the 1 M metal salt solution, the C=O band of GO decreases slightly compared to the C=C elongation. The decrease is slightly visible for GO-Mg, GO-Al and GO-Zn and more importantly for GO-Fe and GO-Sn. This decrease in the ratio indicates a higher level of graphitic material, therefore a higher level of reduction in GO-Fe and GO-Sn hybrid, which is supported by the XPS C 1s spectrum (Figure 2B).

The XPS C1s spectrum of GO (Figure 2B) exhibited two intense bands, one with a symmetrical shape at 284.7 eV (C–C) and one with an unsymmetrical aspect at 286.9 eV (C–O), 288 eV (C=O) and 290.0 eV (O–C=O) [6,61]. The relative intensity of the C–C band at 284.7 eV is strongly influenced by the nature of the metal ions used for the reduction (Sn > Fe > Zn > Al > Mg). The band represents the amount of graphitic material in GO and therefore can be used to evaluate qualitatively the fraction of reduced carbon found in the composites. Small amounts (<1%) of Fe, Zn, Al and Mg were detected in the composites by XPS, which implies that those metal ions form a stable complex with GO. XPS spectra (Figures S2 and S3) show Fe 2p_3/2_ and Fe2p_1/2_ (711 and 725 eV) [62,63], Zn 2p_3/2_ (oxide) (1022 eV) [64], Al 2p (oxide) (76 eV) [65] and Mg 2p (oxide) (53 eV) [66], respectively, for GO-Fe, GO-Zn, GO-Al and GO-Mg composites. For GO-Sn composite, deconvolution showed peaks (0.9 < FWHM < 1.4 eV) corresponding to Sn in different valence state. Peaks located around 485.9 and 494.3 eV could be assigned to Sn^0^ 3d_5/2_ and Sn^0^ 3d_3/2_, respectively; peaks at 486.3 and 494.8 eV to Sn (II, IV) oxide; and peaks at 486.8 and 495.3 eV to Sn (II, V) chloride (Figure S4) [67,68]. According to values found in the literature, GO has a standard potential estimated to −0.4 V (SHE) at pH 4 [54,55]. Because the reduction potential of GO is lower than Sn^2+/^Sn (−0.14 V) [69], GO can supply electron back to trigger spontaneous reduction of Sn^2+^ into Sn metal particles [50,56]. This can explain the trace presence of Sn^0^ in the XPS spectrum (Figure S4). Noteworthy, residual chlorine was detected in small amounts (0.1–0.2%) for GO-Zn, GO-Al and GO-Mg. In contrast, GO-Sn and GO-Fe have non-negligible amount of Cl in their structure and it was estimated to 3.1% and 0.8% by atomic composition, respectively, for the as-dried Sn- and Fe-based GO composites.

The Raman spectra of graphene oxide (GO) and GO decorated with various oxide nanoparticles of Sn, Fe, Zn, Al and Mg are presented in Figure S5. GO have intense D and G peaks at 1350 cm^−1^ and 1592 cm^−1^, respectively, along with a weak and broad 2D band. D and G peaks are the characteristic peaks of GO [6,58,61,70]. The G peak is associated with in-plane bond stretching of sp^2^-hybridized C atoms in both rings and chains. The D peak is due to the breathing mode of sp^2^-hybridized C atoms in aromatic rings. The other five spectra have a similar pattern as the spectrum of GO with slightly blue-shifted D and G peaks. The relative D-band and G-band peak intensities (*I_D_/I_G_*) reflect the density of defects in the sp^2^ lattice [71].

The average crystalline size of the sp^2^ lattice (*La*) of each material can be calculated using the intensities of the D and G peaks according to the following equation [72]:

$$La=2.4\times 10^{-10}\lambda^{4}{}_{laser}(I_{G}/I_{D})$$

where *λ_laser_* is the wavelength (nm) of the laser used for Raman measurements. The calculated average crystalline sp^2^ lattice of GO and reduce GO shows an average value around 22 nm for GO and the *La* values of the reduced GO decreased to 18.1 nm. The different values of *La* for reduced GO reflect the variation of the defects with the sheet sizes.

The as-dried GO-MO composites were not paramagnetic and were characterized using DP-^13^C-SS-NMR (MAS) spectroscopy. ^13^C-SS-NMR (MAS) spectroscopy is a powerful tool to investigate the chemical environment in GO’s structure [61,73] and integration of the signals found in the spectra was used to establish the fraction of reduced carbon (FRC) of the GO-based composites with accuracy [57]. Figure 2C shows the DP-MAS spectra of GO and GO-MO composites. The spectrum of GO-Sn shows a high level of graphitization at 134 ppm and some residual signal at 61 (O–C–O), 164 (O=C–O) and 190 ppm (C=O). The data for GO-Fe, GO-Zn, GO-Al and GO-Mg show the characteristic signals of GO at 61 (O–C–O), 70 (C–OH), 101 (lactol), 134 (C=C), 164 (O=C–O) and 190 ppm (C=O). The fraction of reduced carbon (% C–C) contained in each compound was calculated using Equation (S1) (Supplementary Materials).

Remarkably, NMR spectra show a very smooth differential in the intensity from GO → Mg → Al → Zn → Fe → Sn. Table 2 shows the fraction of reduced carbon (FRC) contained in the composite calculated using NMR integration. The FRC was estimated at 26–75%, which confirms the low level of reduction in GO-MO composites. The method of reduction of GO using 1 M of metal salts proposed in this work gives GO materials with a level of reduction similar to the mild method using bacterial respiration (31–95%), for example [74].

**Figure 3:**
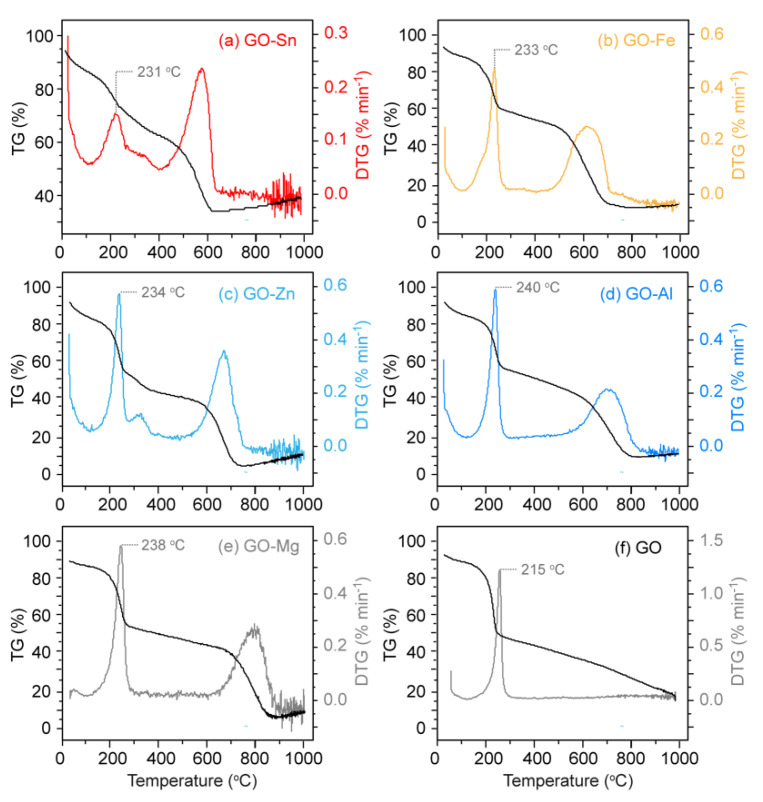
TG and DTG analysis of: (**a**) GO-Sn; (**b**) GO-Fe; (**c**) GO-Zn; (**d**) GO-Al; (**e**) GO-Mg; and (**f**) GO.

As observed in Figure 3, TG and DTGA data of GO and GO-MO composites show two distinct drops of mass: one at 106 °C and a second around 215–240 °C. This first drop indicates a loss of water (4–5%) trapped in the GO materials, while the second drop represents the loss of labile oxygen-containing functional groups leading to H_2_O, which is very useful to compare the level of oxidation among entries [61]. Noteworthy, this second step shows an increasing temperature for GO-MO compared to GO, indicating that the hybrids are more stable thermally. However, around 580–773 °C, a third drop occurs showing the pyrolysis of the GO backbone. A fourth phenomenon starting at 700 °C is observed as a small increase of the weight, which can be attributed to the oxidation of the different metal. Using TGA integration, the second drop of mass shows that GO-Sn has 15.9% of labile oxygen atoms in its composition which is three times less than GO (45%). These TGA results are consistent with the ratio of the fraction of reduced carbon (75% ÷ 24% ≈ 3) obtained from NMR integrations (Table 2). Because water can be tightly bound to GO materials (4–5%), it is difficult to use TGA integration to compare all data with accuracy.

Finally, Table 2 shows that Sn(II) in solution has a greater tendency to reduce GO in ambient conditions compared to other data. According to earlier reports [52,60], a GO-Sn(II) complex is formed in solution and oxidation of Sn(II) to Sn(IV) (E^0^ = −0.77 V) can explain the electron transfer for the reduction of GO according to the following equation:

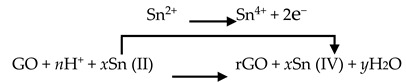


Similarly, we can argue that Fe(II) can also get oxidized to Fe(III) (E^0^ = −0.15 V) [69] in solution after complexation with GO. This can supply one electron for the reduction of GO in GO-Fe composite. Because Zn(II), Al(III) and Mg(II) cannot reach a higher common oxidation state easily in solution, a similar reduction mechanism based on an electron transfer is not plausible. Similar to metal ions, Zn(II), Al(III) and Mg(II) have the ability to form complexes through the oxygenated bonds found on GO’s surface (GO-O-M). In acidic aqueous solution, the GO-OM bond can break, leading to GO losing one oxygen and the metal ion gaining one. This acidic solvolysis-type mild reduction [52] mechanism can be generalized for other metals according to the following equation:

$${\mathrm{GO}+M}^{n+}{\mathrm{Cl}}_{n}\overset{\mathrm{pH}:1–5}{\underset{H_{2}O,r.t.}{\to }}\;\mathrm{rGO}\text{-}\mathrm{MO}+n\mathrm{HCL}$$

## 4. Conclusions

We propose a scalable metal ions-assisted homogenous coprecipitation method for the formation of GO@MO hybrids at room temperature in acidic aqueous solution without any additional reducing agents or physical treatments to promote the reaction. Various analytical techniques including TEM, XPS, FTIR, Raman analysis, TG-DGTA and especially 13-C SS-NMR spectroscopy clearly showed a mild and tunable reduction of graphene oxide materials with a low fraction of reduced carbon and that GO@MO hybrids could be successfully prepared by the process proposed herein. We found that metal chloride hydrates generate their own hydrochloric acid with the reaction of water and GO, which plays a key role in both eliminating oxygen atoms from graphene oxide and assisting certain metal ions to restore the crystal structure of graphene with electrons. We firmly believe that the unique method presented here deserves to be regarded as a cleaner and more economical preparation process for mass production of GO@MO hybrids with moderate level of oxidization and with a better control of their structure at the nanoscale.

## Figures and Tables

**Table 1:** Atomic composition (XPS) of: (a) GO-Sn; (b) GO-Fe; (c) GO-Zn; (d) GO-Al; (e) GO-Mg; and (f) GO materials.

Compound	% C	% O	% M	% Cl	C/O Ratio
GO-Sn	55.4	34.8	6.8	3.1	1.6
GO-Fe	64.2	34.0	1.0	0.8	1.9
GO-Zn	65.1	34.6	0.2	0.1	1.9
GO-Al	65.6	33.9	0.2	0.1	1.9
GO-Mg	64.9	34.4	0.3	0.2	1.9
GO	6	35.7	0.0	0.0	1.8

**Table 2:** Fraction of reduced carbon (%) calculated by NMR and weight loss (%) calculated by TG-DTGA for GO-Sn, GO-Fe, GO-Zn, GO-Al, GO-Mg and in GO materials.

Entries	Compound	FRC (%)	Weight Loss (%)
a	GO-Sn	75	16
b	GO-Fe	38	35
c	GO-Zn	31	31
d	GO-Al	28	39
e	GO-Mg	26	39
f	GO	24	45

## Supplementary Materials

The following are available online at https://www.mdpi.com/2079-4991/10/7/1255/s1, Figure S1: EDS spectra of GO-Sn (up) and GO-Fe (bottom) composites, Figure S2: XPS spectra (Survey) of (a) GO-Sn, (b) GO-Fe, (c) GO-Zn, (d) GO-Al, (e) GO-Mg and (f) GO materials, Figure S3: XPS spectra of (a) GO-Fe, (b) GO-Zn, (c) GO-Al and (d) GO-Mg composites, Figure S4: XPS spectrum and deconvolution of GO-Sn composite, Figure S5: Raman spectrum of (a) GO-Sn, (b) GO-Fe, (c) GO-Zn, (d) GO-Al (e) GO-Mg and (f) GO.
