# Supplementary material for: Room-Temperature Reduction of Graphene Oxide in Water by Metal Chloride Hydrates: A Cleaner Approach for the Preparation of Graphene@Metal Hybrids
Source: Nanomaterials (Basel). 2020 Jun 28;10(7):1255. doi: 10.3390/nano10071255 (PMC7408530; doi:10.3390/nano10071255)
Supplement: Supplementary file 1 [file nanomaterials-10-01255-s001.pdf]

Supplementary Materials

Room-Temperature Reduction of Graphene Oxide in Water by Metal Chloride Hydrates: A Cleaner Approach for the Preparation of Graphene@Metal Hybrids

Patrick. P. Brisebois [1], Ricardo Izquierdo [2] and Mohamed Siaj [1,*]

[1] Department of Chemistry, Université du Québec à Montréal, NanoQAM/QCAM, Montreal, QC H3C 3P8, Canada; patb.office@gmail.com

[2] École de Technologie Supérieure, Université du Québec, Montreal, QC H3C 1K3, Canada; ricardo.izquierdo@etsmtl.ca

* Correspondence: siaj.mohamed@uqam.ca; Tel.: +1-514-987000 (ext. 1921)

Received: 18 May 2020; Accepted: 16 June 2020; Published: date

Equation S1:

$$\%\ \text{C-C} = \frac{100 \times \int^{134\ \text{(ppm)}} \text{C=C}}{\int^{190} \text{C=O} + \int^{164} \text{O-C=O} + \int^{134} \text{C=C} + \int^{105} \text{lactols} + \int^{70} \text{C-OH} + \int^{61\ \text{(ppm)}} \text{C-O-C}}$$

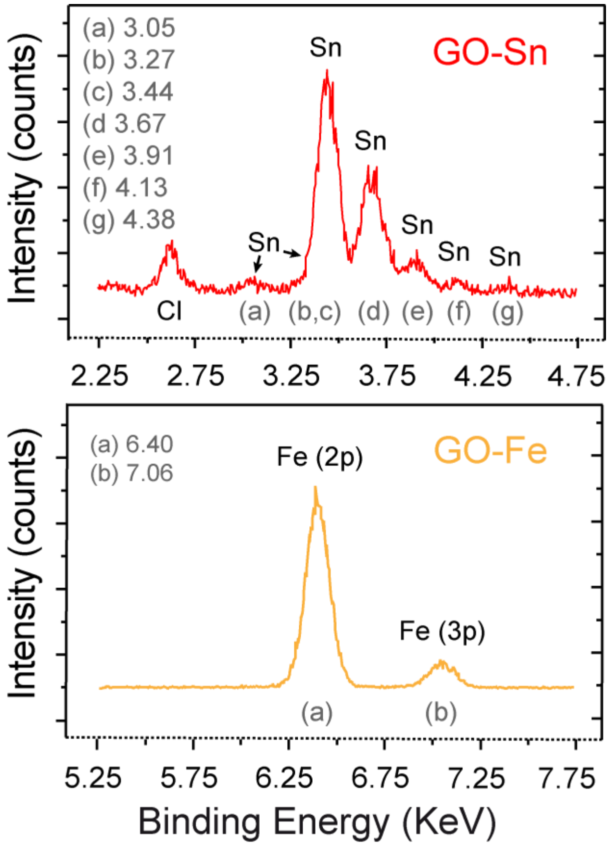

Figure S1. EDS spectra of GO-Sn (up) and GO-Fe (bottom) composites.

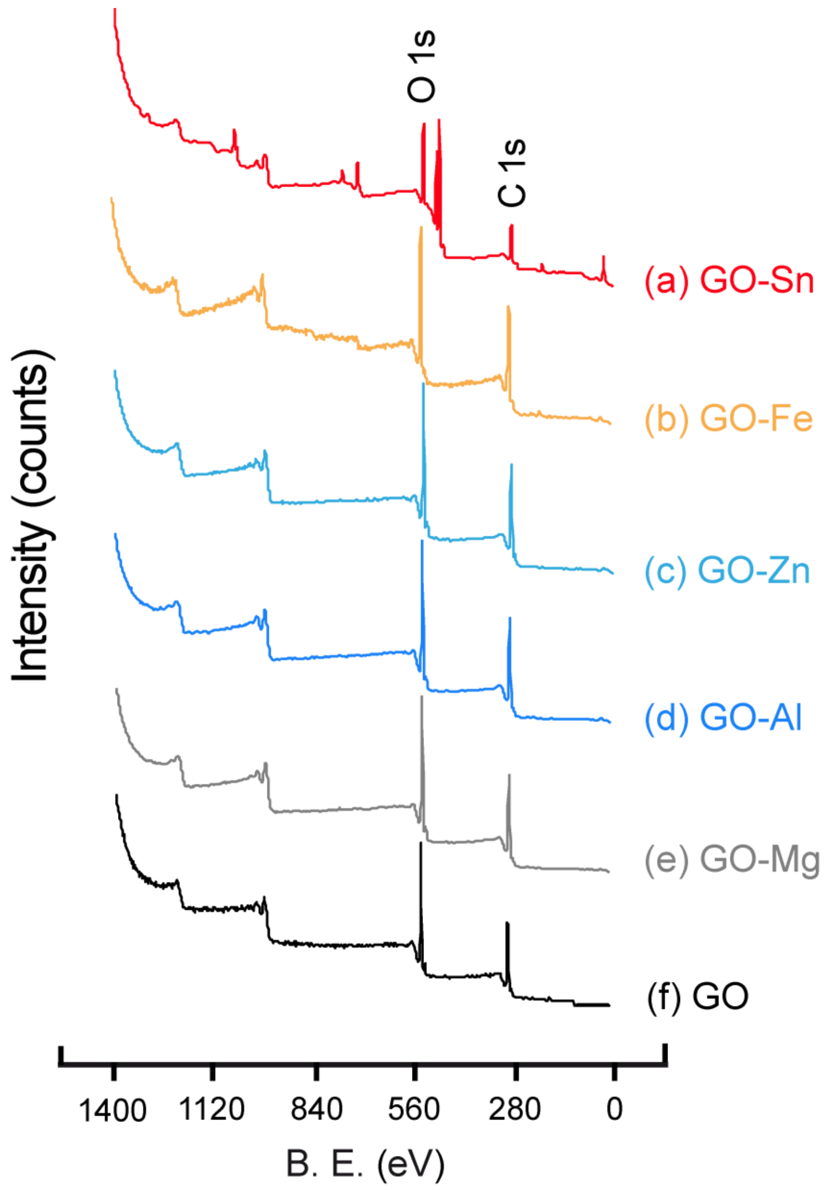

Figure S2. XPS spectra (Survey) of (**a**) GO-Sn, (**b**) GO-Fe, (**c**) GO-Zn, (**d**) GO-Al, (**e**) GO-Mg and (**f**) GO materials.

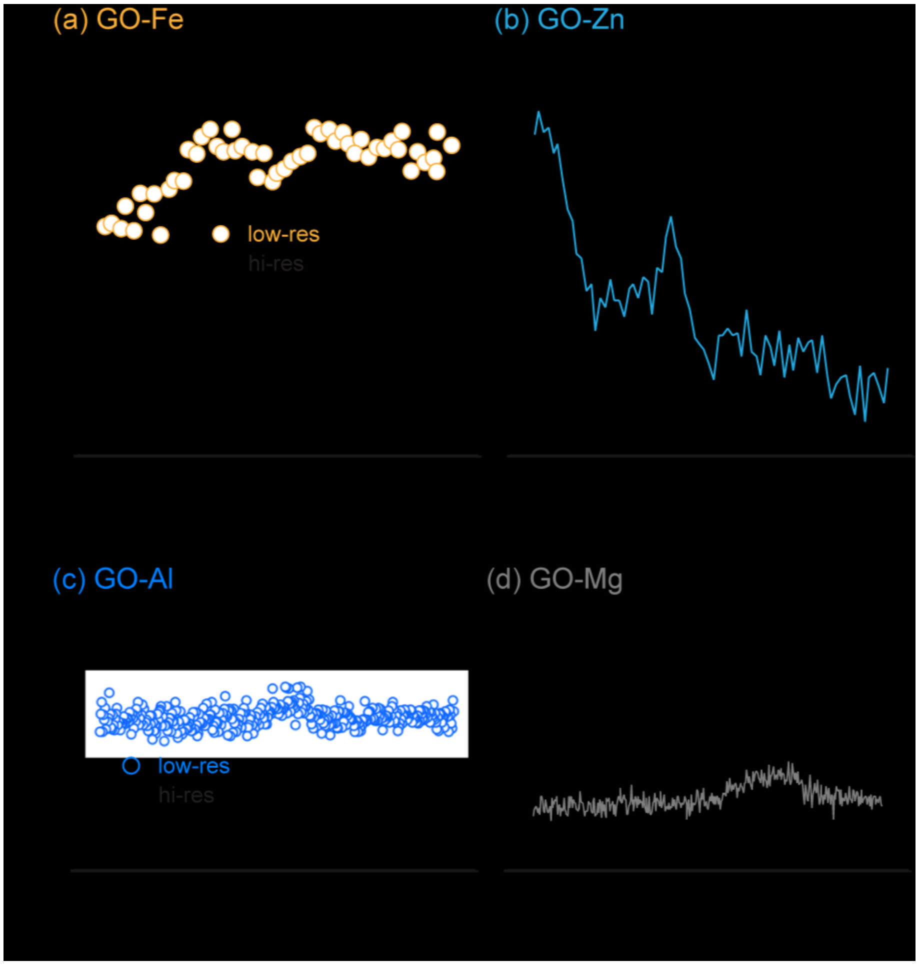

Figure S3. XPS spectra of (**a**) GO-Fe, (**b**) GO-Zn, (**c**) GO-Al and (**d**) GO-Mg composites.

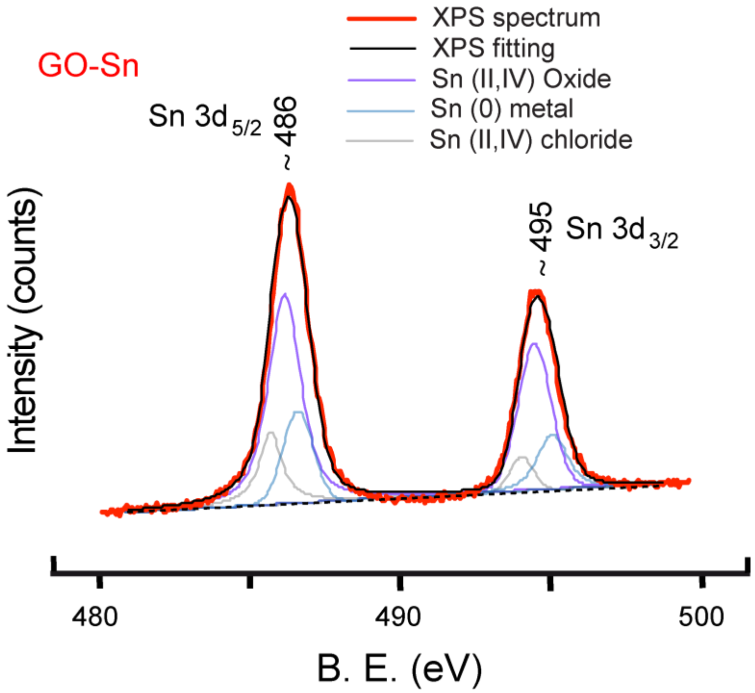

Figure S4. XPS spectrum and deconvolution of GO-Sn composite.

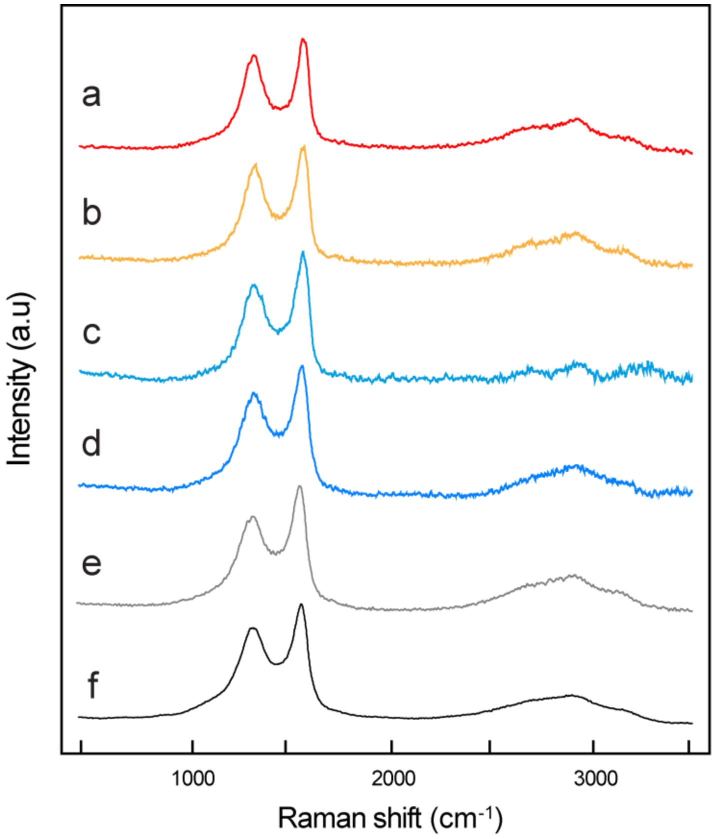

Figure S5. Raman spectrum of (**a**) GO-Sn, (**b**) GO-Fe, (**c**) GO-Zn, (**d**) GO-Al (**e**) GO-Mg and (**f**) GO.
